# Status, identity, and language: A study of issue discussions in GitHub

**DOI:** 10.1371/journal.pone.0215059

**Published:** 2019-06-14

**Authors:** Jingxian Liao, Guowei Yang, David Kavaler, Vladimir Filkov, Prem Devanbu

**Affiliations:** Department of Computer Science, University of California Davis, Davis, California, United States of America; Indiana University, UNITED STATES

## Abstract

Successful open source software (OSS) projects comprise freely observable, task-oriented social networks with hundreds or thousands of participants and large amounts of (textual and technical) discussion. The sheer volume of interactions and participants makes it challenging for participants to find relevant tasks, discussions and people. Tagging (*e.g*., @AmySmith) is a socio-technical practice that enables more focused discussion. By tagging important and relevant people, discussions can be advanced more effectively. However, for all but a few insiders, it can be difficult to identify important and/or relevant people. In this paper we study tagging in OSS projects from a socio-linguistics perspective. First we argue that *textual content per se* reveals a great deal about the status and identity of *who is speaking* and *who is being addressed*. Next, we suggest that this phenomenon can be usefully modeled using modern deep-learning methods. Finally, we illustrate the value of these approaches with tools that could assist people to find the important and relevant people for a discussion.

## Introduction

In distributed software engineering, people work both individually and in teams to complete programming tasks, using collaborative platforms such as GitHub. These platforms support social interactions using mechanisms such as tagging [1]. As an example, a user named *evaphx* uses “@” tagging to invoke another user *djones*, to help with issue 1189 in the puma project [2]:

@djones Just to confirm, websockets not over SSL work fine?

This is the norm in modern collaborative development. Developers call on others, and get called upon, to contribute their expertise whenever the need for it arises. Thus, someone can be called to contribute to an issue discussion regarding a feature or a bug, if they’ve previously been involved in the development of related code.

Online discussions in software projects are critical and non-trivial, and are also voluminous. Tagging is an important mechanism to help streamline collaboration, but must be done with care. Often people who are tagged to contribute to a task are responsive, but sometimes are not, especially if the task is not relevant to their skillset. Scattershot, futile tagging of people who fail to respond can lead to wasted time. In many open source projects, both expertise and productivity matter; most of the work gets done by a few people [3], and getting the right people involved helps sustain quality [4]. In large projects, however, it may not always be easy for developers, specially new developers, to know a) who to tag for a specific task, or b) even who the most relevant and engaged people in the project are. Identifying the right people to tag, therefore, becomes an important task, one that to our knowledge has not been properly addressed.

Our goal here is to explore the possibility of helping developers with these challenges using a linguistic perspective. We hypothesize that *the language people use to communicate* provides strong clues about their productivity and social status. Linguistic cues for a person’s status and productivity may be latent in *the way that person speaks*, and also in *the way that person is spoken to*.

Software projects on GitHub contain voluminous and publicly available data on issue discussions, containing both socio-linguistic information (how you address people, and how you speak) and status and productivity information (how you commit). Starting from a data set comprising discussions in hundreds of GitHub projects, we seek in this paper to evaluate how *language is associated with the speaker* and *language associated with the person who is tagged*, per se, can predict the status of the person who is speaking or is spoken to. As explained below, our work thus is both a scientific interrogation of whether the language used in these dialogs is indicative of status, and an evaluation of the possible utility of language cues to facilitate such dialog.

The linguistic data in issue discussions is very high dimensional and heterogenous, containing both general vocabulary words and project-specific idiosyncrasies, and is thus challenging to digest. We apply deep learning to process and contextualize posts, in particular we use representation learning, via stacked de-noising auto-encoding [5], that has been proven effective in learning representations.

## Theory and related work

Social discussions in software projects are sizable, highly technical, and important for project success [6–8]. In GitHub, the norm is to use *@-mentions*, similar to a “tag” in other social networking systems. By @-mentioning another user, one can, *inter alia*, call their attention to a particular issue [9, 10], to get feedback, or help with a task-related action. The idea of assigning tasks to appropriate individuals has been discussed extensively in Software Engineering, especially in bug [11, 12] and pull request assignment [13, 14]. Proper assignment is of great importance, as it has been shown that a minority of individuals do most of the work in open source projects [3]. Thus, it follows that a system which can automatically identify the most relevant and responsive individuals would help developers, specially during on-boarding [15].

From a practical, tool-creating standpoint, given that productivity information (through, *e.g*., git histories) is readily available in these projects, a simple tool which identifies those with the highest productivity would be trivial to create. However, in this work, we are interested in studying the usage of *language* in open source projects. Specifically, is it possible to identify highly productive and/or highly relevant individuals solely by the language they use in GitHub issue discussions?

Prior research supports this possibility. Project-specific dialects have been shown to emerge in open source projects, with project teams gradually drifting towards linguistic norms [16]. Status-specific attention and address has also been observed: social psychologists have found effects of *enclothed cognition*, *e.g*., individuals who wear a white lab coat, described as a coat worn by doctors, have increased sustained attention [17]. In addition, wearing formal clothing has been shown to be associated with higher action identification level and greater category inclusiveness [18]. These works serve as a psychological basis—theoretically, individuals who perceive themselves as having a particular social status may address others differently, and may likewise be addressed differently. Research in the field of sociolinguistics further supports this notion. In the 1960s, Labov showed that social and class aspirations influence speech patterns; those wishing to be associated with a certain class will adjust their speech patterns to sound like those they aspire to be [19]. In addition, researchers have shown that situations in which a power difference exists between individuals will result in hierarchical differentiation of language use [20]. For example, Kacewicz *et al*. [21] found that pronouns are used differentially by individuals based on positions within social hierarchies; those with higher status consistently used fewer first-person singular, and more first-person plural and second-person singular pronouns. More directly related to our work, Dino *et al*. [22] found that low status members on Internet message boards used more first-person singular voice, affective words, and exclamation marks, while high status members had messages that were more instructive and contained more complex words, second person references, and welcoming language.

Differential association of language with social status also has practical relevance. People communicate using online forums for a range of different reasons: simple socializing, discussing specialized topics (politics, science, etc.), use & maintenance of complex items like pets, plants, computers, software, or automobiles, or to organize social activities. In all these communities, identifying high-status individuals can be very useful, both for individuals seeking authoritative opinion, and for businesses seeking to market products or recruit high-skilled or influential individuals. However, identifying status of individuals in very active groups is complicated by volume, and the (sometimes deliberately obscurantist) use of aliasing. In such settings, a reasonably accurate way of identifying the status of individuals, solely by the way they speak (or are spoken to), without knowledge of their identity, could have practical value.

Given this theoretical support from social psychology and sociolinguistics, and potential practical value, we seek to see if the language used in GitHub issue discussions, *per se*, can be leveraged to identify highly productive individuals. The advantages in the GitHub setting are: a) there is a large amount of linguistic data, and, b) the linguistic data can be *independently, reliably, and cheaply labeled* using commit histories in the source code version repository. Thus, we can use this data to study the extent to which status (or topical relevance of an individual) is derivable, solely from the linguistic manner of address and speech.

In this work, we do not profess to fully clarify deep sociolinguistic or psychological reasons behind the stratification of language within the social and technical hierarchy of GitHub. However, we do provide some initial evidence that such stratification exists, specifically by building models which can identify highly productive contributors (and even specific individuals) based only on the language they use and the language used when speaking to them.

## Data and methodology

We sought data relevant to two tasks: first, identifying the highest status (*viz*, most active committers), and second, identifying individuals to be called upon—both, *purely just* from they way they speak or are spoken to. All our data were collected by using the Python package PyGithub [23] through the GitHub public API. We randomly sampled 50 projects from the top 900 GitHub projects with the most stars and followers, which reduced to 46 after removing those having missing data. We chose the top 900 projects to ensure there is a sufficient amount of text in their issue communications; we chose to sample in order to a) reduce the amount of time it takes to build our models; and b) avoid bias that may exist when examining only the very top projects.

We gathered all comments available in the issue threads and commit records for the 46 sample projects, from their inception date to March 2017. For each comment, we collected the text, date, login account of the poster, the thread number, and the closing date of the issue. If the thread was still open, the closing date was omitted. For every commit record we gathered: the lines of code added and deleted, the commit date, and the code author and committer. Note that the code author and committer may be different in principle; however, when reviewing our data, we found that in the majority of commits, the code author and committer are the same.

In summary, our dataset includes 11, 046 developers who committed to a project, and 44, 161 participants who joined the discussion in the issue threads, for a total of 464, 793 posts, with 163, 789 containing at least one @-mention. Even though all the projects are popular, the committer count in each project ranges from dozens to thousands. The heterogeneity between projects will be considered later in the discussion section. When testing the learned models, the @-mention names are replaced by generic @ tokens to combat information leakage from training to testing, while maintaining the grammar of sentences. Precise reasons for these modifications are explained below.

### Data cleaning and processing

Posts in the GitHub issue threads are quite different from ordinary social communications and require special cleaning and filtering. Developers talk about project bugs, enhancements, and tasks in issue discussions [24]. As a result, this corpus includes plenty of code, warnings, messages, and technical terminology in addition to more general natural language. This may provide very domain-specific, idiosyncratic vocabulary cues (*e.g*., file names or method names) which allow for easy detection of status and/or relevant expertise. Since we are interested to see whether *general language* provides indications of status and/or relevance, we filter out the code-specific bits. Fortunately, these are discoverable through code insertion HTML markups, i.e., <CODE >…<CODE >, within our corpus. Thus, we replaced everything within those markups with the token <CODE_TOKEN> so that what remains resembles normal social communications.

We also found that short posts, having fewer than 5 tokens, have scant information about projects and posters. Most of them are appreciation or closure of an issue, such as *“Thanks @apfritts”* in the project *jigish_slate*. These comprise about 13% of the total; we removed them, which still left us 404, 210 posts for analysis. During the preliminary data exploration, we also found that large numbers of posts are posted by developers who never commit code to the project. The rest, about 266, 483 posts, or 57.3% of the whole dataset, were made by committers. When distinguishing between very highly productive developers and the rest, the non-committers will have a value of 0 for productivity, and thus inflate the number of zeros in the data, i.e., cause *zero-inflation*[25], which may confound the evaluation of model performance. Therefore, we have two pairs of datasets for that task: one including posts from all developers, and posts including @-mentions of anyone (denoted *“full dataset”*); and another comprising only posts by project committers and posts only having @-mentions of just these committers (denoted *“committer-only dataset”*).

We assembled a social network graph from the @-mentions in posts. The network is a directed graph, where each edge (*u*, *v*) represents one @-mention from developer *u* to developer *v*. We also include “weights” on the edges, as the count of times that this @-mention is observed. Note that in such a network, the metrics of node *indegree* and *outdegree*, respectively correspond to the social measures of developer popularity (who tagged ego), and familiarity (which alters did ego tag) with the rest of the network.

Next, from the issues and commit data, we also calculated other features of developers’ activities: *tenure*, *commit count*, and *comment count*. The count of comments is a straightforward measure of developers participation. We estimated developers’ tenure in a specific project by their activity duration. Developer participation starts when they first post in any issue or change commit, and ends on their last post or commit. The same developer participating in multiple projects is considered as an independent developer in our models, since most projects are disjoint in their topics; this situation is also rare in our data.

**Fig 1:**
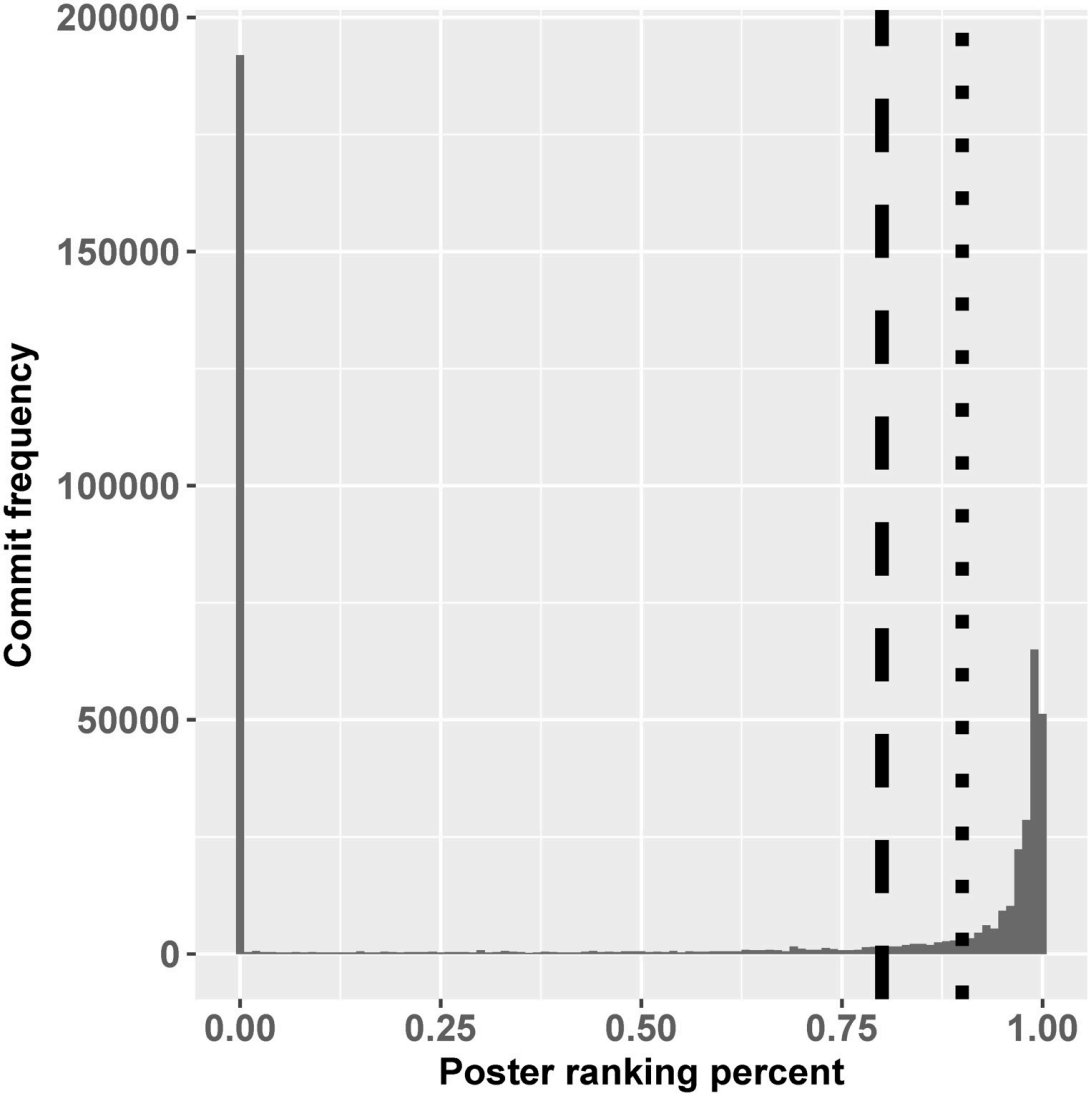
Histograms of poster ranking based on commit frequency. The dashed line indicates top 10% cutoff; dotted line indicates top 20% cutoff. Although population sizes are different the distributions follow a very similar pattern. The bin at zero on the left contains all the non-committers.

Next, for every developer, we count the number of commits they have made over their tenure. We define “highly-productive” developers as those whose commit count lies within the top 10% of all committers’, for each project. This percentage was chosen empirically. Our preliminary exploration indicates a natural gap between these developers and others. Fig 1 illustrates this gap. On the *x*-axis, we show the percentile (1.0 = 100%) ranking of developers, with the most productive positioned rightmost. On the *y*-axis (“Commit frequency”) is the number of commits. The dotted line suggests that 10% is an appropriate cut-off between the most frequent and the rest; the dashed line represents a 20% cut, which is less significant. Combined with the non-committers discussed above, we split developers into three groups: the top 10% committers, other committers, and non-committing end-users. In the full dataset all three parts are included, while the committer-only dataset contains only posts of the top 10% committers and other committers. The ratio between posts of top 10% committers to two other groups is 58 : 42 in full dataset. And in committer-only dataset, the ratio of posts of the 10% committers to posts of other committers are 25 : 75. Given the rather imbalanced dataset, we use the non-parametric AUC method for evaluation, as discussed below.

### Language models

In this section, we describe the use of language models (LMs) built to predict developers’ productivity, specifically, to classify developers as “highly-productive” or not (binary classification). The LMs are under two scenarios: predicting developer productivity level from their posts, *i.e*., their *spoken language* (SpeakingP), and predicting developer productivity level from posts in which they are @-mentioned, *i.e*., *spoken-to language* (SpokenP). All models were built using the Python package Keras, over a Tensorflow back-end. Technical details follow.

We first tokenized the post texts using NLTK’s TweetTokenizer [26]. After tokenization, samples become token sequences of varying lengths. For some of our experiments, as described below (where we evaluate whether sequence lengths alone is enough to predict developer status or relevance) we pad or truncate the sequences to be of fixed length.

The LMs a five-layer deep network: a word embedding layer, one-dimensional convolution and maxpooling layers, a Long-Short-Term Memory (LSTM) layer, and a sigmoid dense output layer. Word embedding is a standard way to map word context into a continuous lower-dimensional vector space (widely used in a range of applications, such as question answering [27], machine translation [28]). We use the Glove [29] (Global Vectors for Word Representation) embeddings. The convolution layer uses data windows to process local parts of the input data, while the max-pooling layer collects a single representation by maximizing a set of neighbors. They capture and summarize the meanings in the text input. This is fed into an LSTM layer, a standard technique for text processing [30]. It comprises 30 hidden cells with ReLU activation. The last layer is a fully connected sigmoid output layer. This dense layer makes sure the output is a probability, i.e., in the range [0, 1]. Binary cross entropy is used as the loss function. All layers are trainable during the whole training process, *i.e*., and the word embeddings are not static. We have shared all the code and detail about language models in the GitHub repo mentioned in the supporting file, with a tutorial of implementation.

The Speaking Developer Productivity (SpeakingP) task is to predict whether or not the speaking developer in an issue is within the top 10% of all committers, using just the textual content of their post (for posts longer than 5 tokens). For this task, we train the entire network by back-propagation, including the embedding layer.

For the he Spoken-To Developer Productivity (SpokenP) task, we try predict the productivity of the person who is @-mentioned. As the spoken-to model is meant to predict the productivity of the @-mentioned user *given surrounding context*, the input data for this model must not contain the @-mentioned login name; otherwise, it would be considerably simpler, as this information would leak from training to testing. Instead, we augment our existing spoken-to productivity model with *contextual* embeddings of @-mentions from a training set, and then use these embeddings in the spoken-to task, to learn productivity levels. Theoretically, the additional information may improve our prediction performance. To do that, we use a specialized model, inspired by sDAE (thus “sDAE-like”), discussed below.

### The sDAE-like model

**Fig 2:**
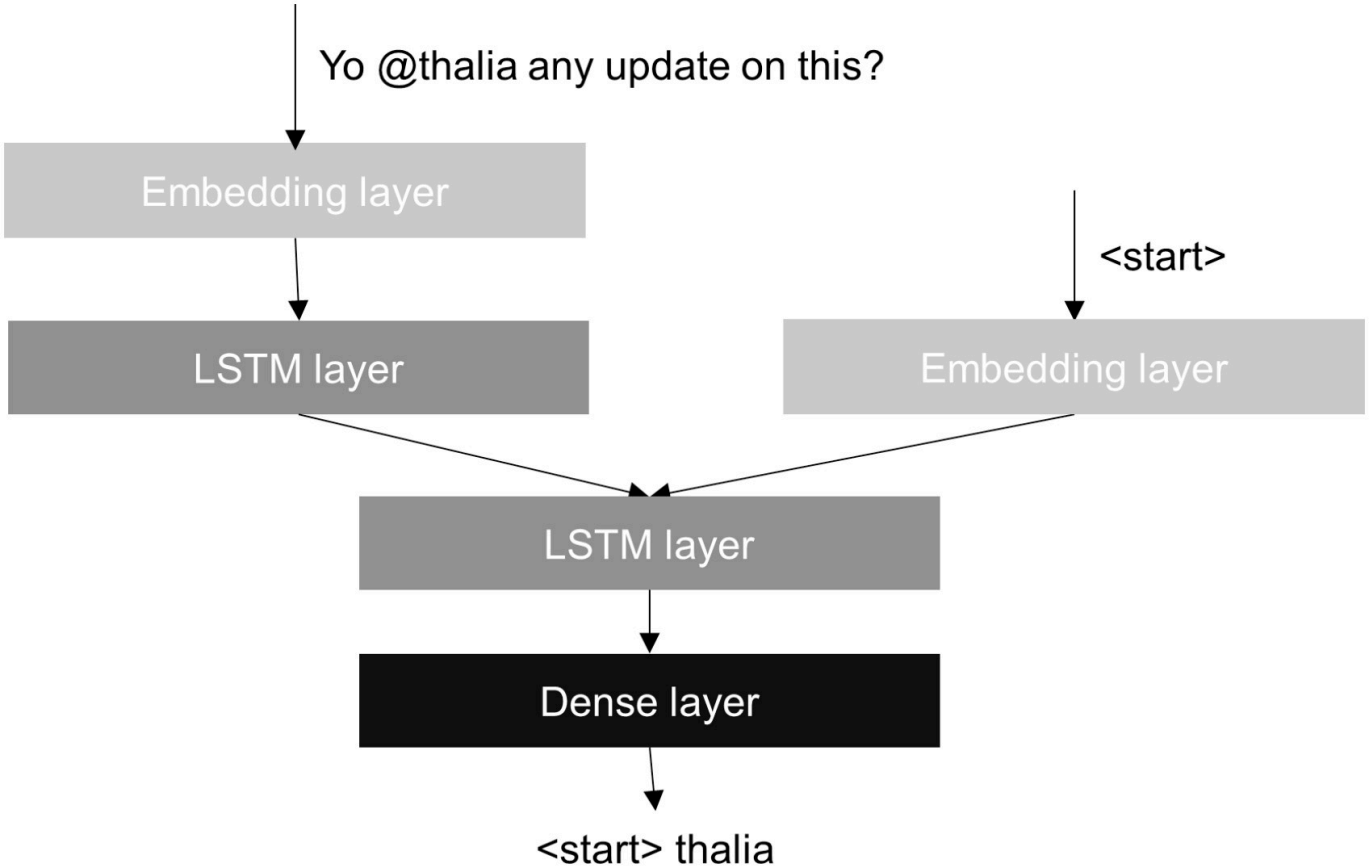
Diagram of the sDAE-like model.

We use a “de-noising” style model to predict the @-mentioned user login name, using contextual similarity as the basis. This model is illustrated in Fig 2 [5]. The general approach of sDAE (*stacked de-noising auto-encoder*) is to learn a representation that gets used within a sequence to sequence model to *de-noise* artificially distorted data: the goal is to reconstruct the original signal given a noised version. This approach aims to learn a representation that captures the statistics of the data sufficent for a highly challenging task (de-noising), from abundant *unlabeled* training data. These sDAE models are trained with artificially “noised-up” samples, for the task of mapping them to the de-noised (original) result. In our case, the original signal is the post with the @-mention; and the noised signal is the post without @-mention. Therefore, our focus is on the @-mention name – the missing part—and the “de-noising” task is to just recover only the login name (not the full post). Our “sDAE-like” model uses LSTM layers in the encoding and decoding phases respectively. This auto-encoder learns a representation of the text, for the purpose of recovering missing @-mentions. This specific goal prompts the contextual embedding layer to learn a representation specially tuned to recover the identity of the missing tagee (@-mention name) from the textual context. This hard task forces the de-noising auto-encoder to learn a good representation that could transcend the generic Glove technique, *for tasks* relating to the people involved in the social interaction. In Fig 2, we start with a “noised” input without the tagee “Yo, @, any update on this?” and the task is to recover the name of the tagee, in this case @thalia.

#### Training and deep learning meta-parameter details

Our language model is a sequential model implemented using Keras in Python 3. The embedding layer has an encoding dimension of 64. Then we have the convolution layer with 6 as kernel size, followed by a pooling layer with 5 as pool size. The LSTM layer has an output dimension of 30. The dropout rate is 0.2. We used “RELU” as activation function for convolutional layer and LSTM layer, and “sigmoid” for the output layer. We choose binary cross entropy as the loss function and “Adam” as optimizer.

The sDAE-like model is an autoencoder model, also implemented using Keras in Python 3. The encoder has an embedding layer and a LSTM layer. The decoder also has an embedding layer and a LSTM layer, plus a “SoftMax” layer as output. We chose a 200-dimensional embedding layer. We used categorical cross entropy as loss function and “RMSProp” as optimizer. We trained our models using a single Nvidia GTX 1080 Ti GPU. It takes less than 3 minutes to train the language model, and it takes 10 to 20 minutes to train the sDAE-like model.

## Results and discussion

In this section we examine the utility of various language features, to evaluate the performance of our LMs, and discuss the prediction accuracy and developer attributes for the sDAE-like model.

Our LMs are binary classifiers, designed to classify the speaking or spoken-to developers’ productivity as being in the top-10%, respectively the bottom-90%, in terms of commit count. The area under the receiver operating characteristic curve (AUC) is the main measure criterion for this classification problem. AUC has a range of [0.5, 1.0], where an AUC of 1.0 implies a perfect prediction, and 0.5 is a random coin flip. We use AUC here, rather than precision-recall or F1 scores, since this is an imbalanced dataset; for such datasets, AUC is a good way to measure improvements over just random guessing. In the following tests, the respective datasets are split into a 70 : 30 ratio to create training and test sets.

As shown in Table 1, the AUC for the LM in the SpeakingP task is 87.02% for the full dataset, representing the discriminatory power between the high- and low-status developers. The AUC in the SpokenP task is 81.06% for the full dataset. For the committer-only dataset, the AUC in the SpeakingP task is 78.44%, and 75.04% in SpokenP. The lower AUCs for the committer-only dataset (*vis-a-vis* the full dataset) suggests that committers are more difficult to distinguish between (than are all developers) using just the language used in the issue thread to call on them. This suggests that @-calls to committers use somewhat different language than calls to general users. We note that the committer-only dataset has a smaller training dataset than the full dataset. To test whether the prediction difference is due to dataset-size differences, we down-sampled the full dataset and re-ran the experiment. As shown, controlling for training set size did not alter our results much. This suggests that the committer-only task is inherently harder.

**Table 1:** AUCs of our language models.

Tasks	Word Embedding	Full Dataset	Full Dataset Downsample	Committer-only Dataset
SpeakingP	Glove	87.02%	86.02%	78.44%
SpokenP	Glove	81.06%	80.61%	75.04%
SpokenP	sDAE-like	80.91%	79.72%	76.02%

Table 1 also includes the AUC for SpokenP with the embedding layer from the sDAE-like model. Due to limitations of memory and computation time, we use the top 15, 000 words, based on word frequency, to perform the sDAE-like model training. We note that the results using embeddings learned in the sDAE-like model are close to those using trainable Glove representations.

### Important features in the language model

Deep learning approaches often yield very effective predictors, but understanding which features are responsible for the good performance is challenging. Here, we evaluate the effect of various non-content-related linguistic features that the model might be learning. If these features play too strong a role, this may be an indication that it is not post content that determines status, but rather that our models are learning something less interesting (*e.g*., post length).

#### Post token-length

**Fig 3:**
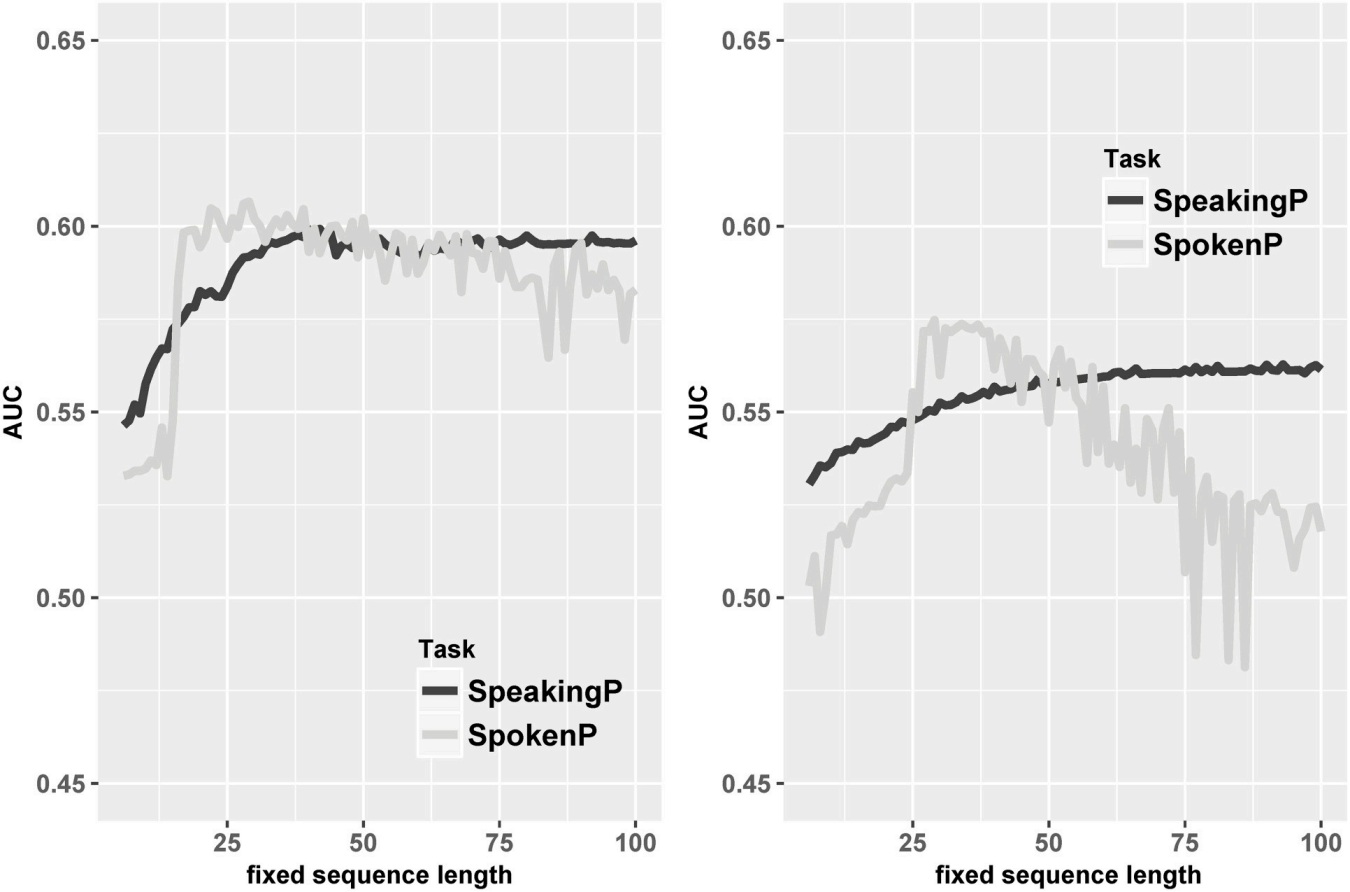
Line plot of the AUC performance of our predictors (SpeakingP and SpokenP) when posts are restricted to having length information only, for different token sequence lengths, for the full dataset (left) and committer-only dataset (right).

First, we consider the length of posts, a simple variable that might be determinative. Prior work has claimed [31] that an average of 15 to 20 words is effective in certain types of technical communication; thus it might also be sufficient in our setting. To evaluate the influence of length *per se*, we padded or truncated the posts to varying lengths, of size *k*, and then learned the models each *k* value. If a post’s length is shorter than *k*, it gets padded with a token (absent from the corpus); if it is larger than *k*, we keep only the first *k* tokens, reading from left to right. Then all the original words are replaced by another token, absent from the corpus; this pre-processing step retains nothing but the token-length. We train and test our models for various values of *k* for this dataset, where each post consists of only two tokens. Results are shown in the Fig 3, with the full dataset on the left, and committer-only on the right.

Here, the dark lines show the AUCs of SpeakingP as fixed post length increases, while the light lines correspond to SpokenP. Generally, when the sentence is longer, it has more information, which should lead to better performance. From the left figure, we see a significant increasing trend as the post length grows, until about 40 tokens in length. Then, the AUC shrinks to about 60%, within a small range. This illustrates that most posts do not contain additional information past token 50 that the LM can use. The light line shows a less stable effect of the length *k* in SpokenP. The basic tendency stays the same, but the AUCs seem extremely volatile when the length grows to more than 75 tokens. The data suggest that the extra tokens increase the noisiness of the data, and lead to unhelpful variance in the vector representations. For committers-only (right), the fixed sequence length is not a very useful feature for SpeakingP. The dark line in the right plot is almost a horizontal line, which keeps the trend of dark line in full dataset. On the other hand, the AUCs for SpokenP resemble the shape on the left, but the performance loss after 50 tokens is more distinct.

We did this study to diminish the possibility that our model performance (shown in Table 1) arises vacuously from just the token length. The above results suggest that this is unlikely, since the length-only models perform a lot worse than the full text models.

#### Syntax: Closed category words

Words in most natural languages fall into two basic categories, “closed” and “open” category words: “closed” are the categories of words that don’t expand very much at all, *e.g*., conjunctions (*and, or*), articles (*the, a*), demonstratives (*this, that*), and prepositions (*to, from, at, with*) [32]. The open category words are all the rest. Closed-class words are syntactic markers; open-class words tend to convey the bulk of concepts and semantics. In this study, we sought to examine whether our classification performance arises mostly from structure, as indicated by only closed-class words, rather than information content within the more expressive, open-class words.

**Fig 4:**
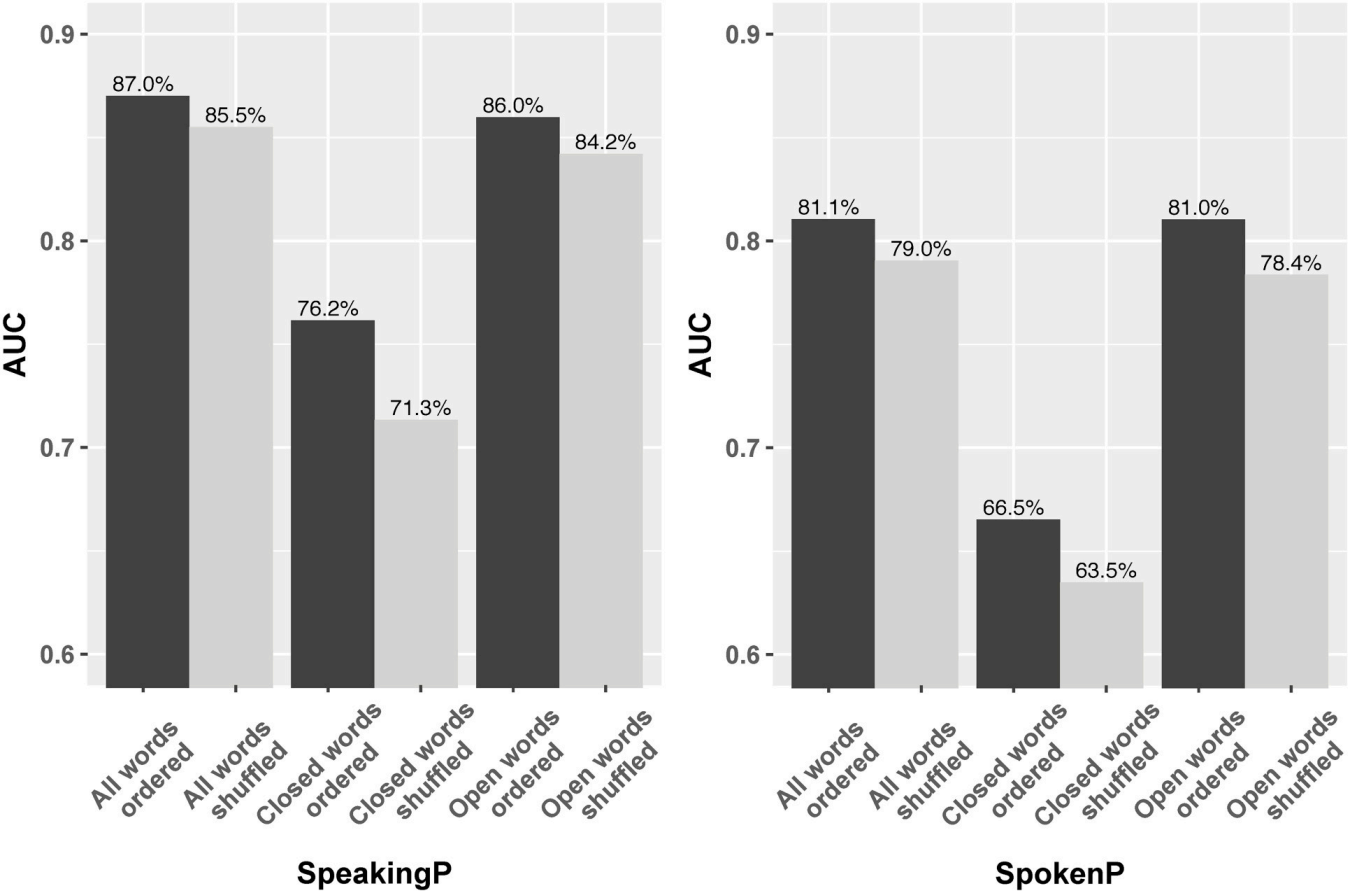
Barplot of AUC comparison for word categories and word ordering in the full dataset.

**Fig 5:**
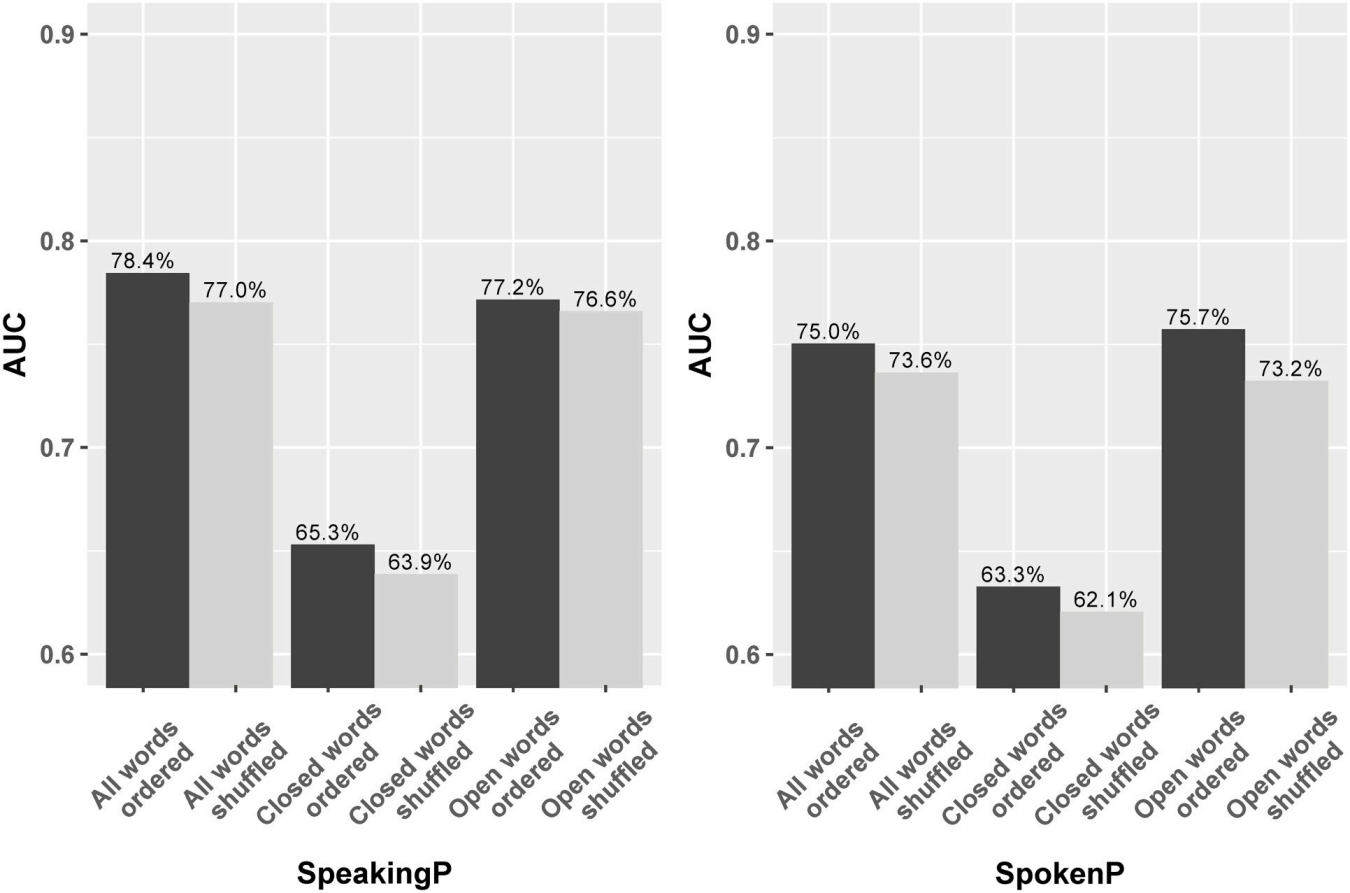
Barplot of AUC comparison for word categories and word ordering in the committer-only dataset.

To do this, we collected a set of 307 common closed-class words online [33] and filtered our input data to retain only these words, replacing all others with a special token (different from the padding token), and vice versa for a secondary test of the performance based only on open-class words. The performance of these methods is shown in Figs 4 and 5. In SpeakingP (left), the AUC using only closed-class words is 76.2%, 10% lower than the original (all words); when using only open-class words, the AUC is almost the same (these values are stable across folds in ten-fold cross validation). As compared to using only length information (60%) (Page 12), structural information seems to provide a significant increase in prediction performance. However, our LM is not only learning structure; the performance of only open-class words is 86.0%, close to the performance of using both word categories. This suggests that the SpeakingP LM relies more on the semantic information contained within open-class words. For SpokenP, their performance trend and differences look similar as in SpeakingP. The LM AUC for the closed-class words only is 66.5% in SpokenP while that for all words is 81.1%. On the other hand, the LM AUC for the open-class words is nearly 81%, almost the same as the one with all words. The committer-only dataset follows the same pattern, shown in Fig 5.

#### Word ordering

Word ordering is vital in many language tasks, such as text generation and machine translation. Languages are constrained by grammatical and semantic rules that predicate word order [34]. Still, quite a bit of semantics resides in the words themselves, regardless of order. Many useful algorithms rely on bag-of-words, which ignore word ordering. Our LMs do preserve and can learn word ordering; we study next the extent to which this matters in the models performance.

To that end, we shuffled the order of words in the entire post, before they are used in the models. Figs 4 and 5 present the outcomes of shuffling word order. Generally, LMs performance decline by only 0.5%–2% on average, after shuffling the words. And the impact of ordering is consistent across multiple experiments and tasks. This implies that reordering the whole sequence before or after the fix length operation has little to do with the final prediction accuracy.

The irrelevance of token ordering means that an important feature of the LSTM layer is not significantly used in our models. The Bag-of-Words method is well known for contexts where ordering does not matter in NLP tasks. So we also applied a BoW model for the same datasets with a multinomial Naive Bayes method as comparison. Because of the very large size of the natural corpus, we keep only the 10, 000 most frequent tokens. The results are in Table 2. Comparing them to Tables 1 and 2, our LMs perform notably better than BoW, as the LMs show about 10% of an AUC advantage. This suggests that the more complex, learnable formulae used by LSTMs to combine constituent word representations provides an advantage over simple BoW.

**Table 2:** AUCs of the Bag-of-Word models.

Tasks	Full Dataset	Committer-only Dataset
SpeakingP	74%	72%
SpokenP	66%	65%

#### Common words

Linguists have long known that the distribution of words in text is highly skewed. The 25 most common words in English constitute more than 25% of all printed material [35]. These words are critical to structure and meaning. In this section, we explore whether our performance is mainly attributable to these words, or not.

**Fig 6:**
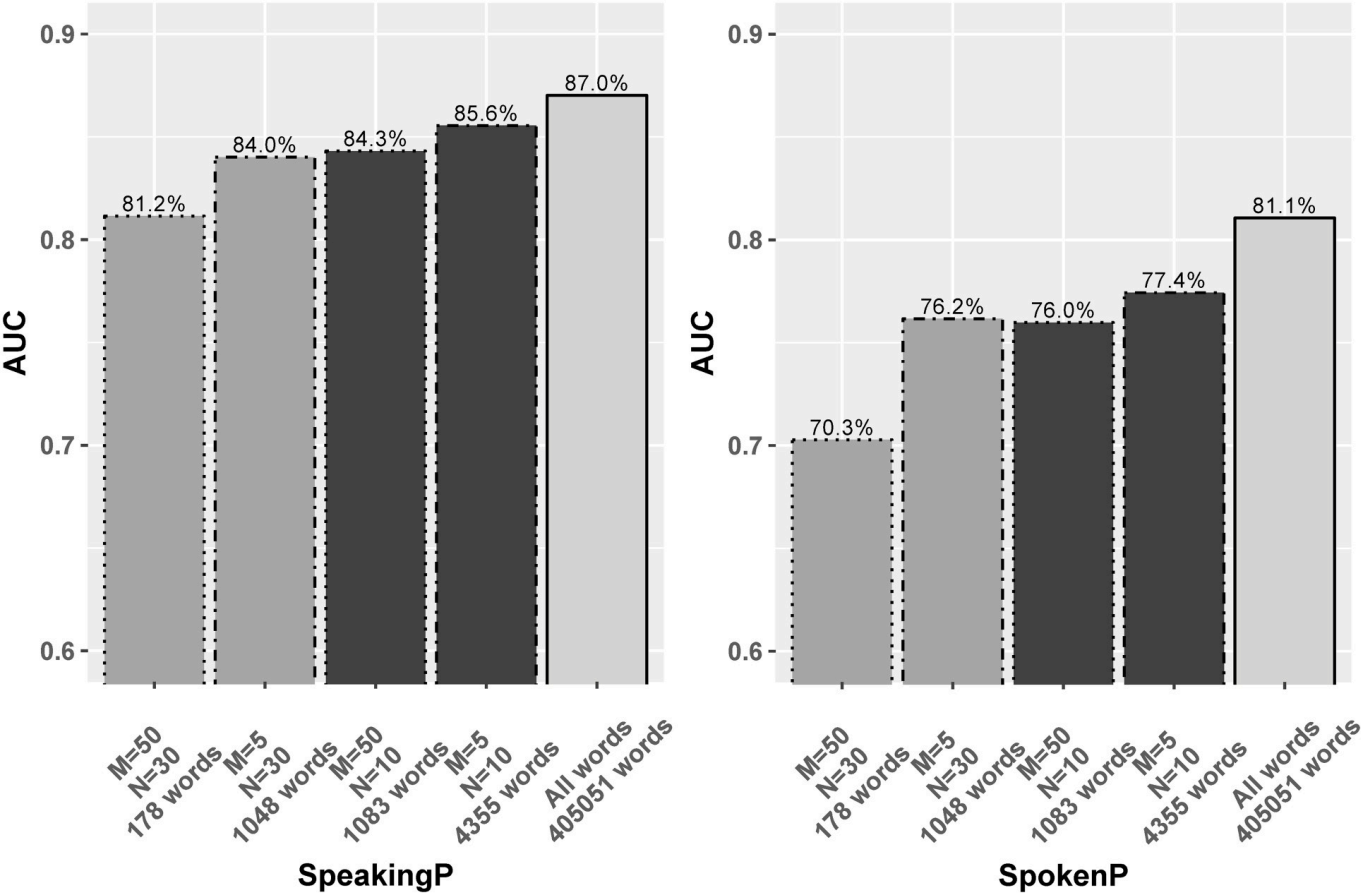
The LMs AUC performance on word sets occurring at least M times in at least N projects in the full dataset.

The range of common words has evolved through the centuries [36], which leaves us the space of building up this small list of common words based on our corpus. Our corpus has over 400,000 individual words (1-grams), and a census shows that the top 100 most common distinct words comprise 50%–60% of words (depending on the project). We define the “common vocabulary set” (CommonSet) as those words occurring at least *M* times in a minimum of *N* projects. As *M* and *N* vary, the common vocabulary varies. We created four sets of words for performance comparison, illustrated in Fig 6. The smallest common vocabulary set is of size 178, denoted as *CommonSet 1*, corresponding to *M* = 50 and *N* = 30. Those account for 58.4% of words, on average, in each project. The 1048 words in *(CommonSet 2)* correspond to the choice of *M* = 5 and *N* = 30. *CommonSet 3* arises from *M* = 50, *N* = 10, and consists of 1083 words. Finally, the 4355 words in *CommonSet 4* are selected based on *M* = 5 and *N* = 10.

For the model based on words in *CommonSet 1* only, the AUC for SpeakingP is 81.2%, a drop of about 6% compared to the full corpus; the AUC for SpokenP is 70.3%. For SpeakingP, the prediction performance grows smoothly with the size of the common words sets. For SpokenP, the AUC increases with the common word set size as well, however, there’s a larger gap in the performance between the smaller common sets and all words. This suggests that our models mainly learn from the information carried by the common words, though that’s much more the case for SpeakingP. The LM in SpokenP relies much more on less common words to identify developers than the LM in SpeakingP, underlining that the two models may rely on different features.

**Fig 7:**
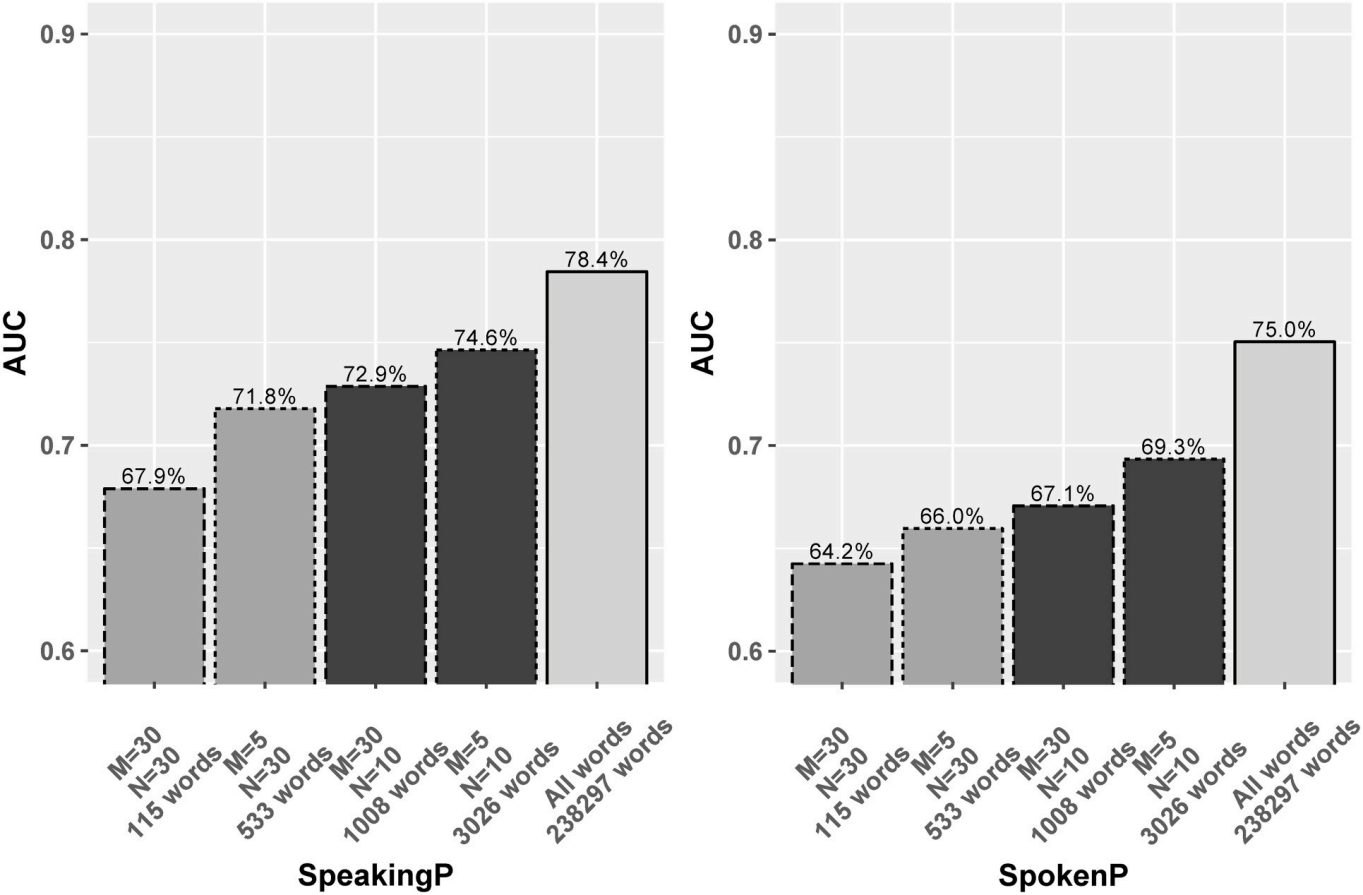
The LMs AUC performance on word sets occurring at least M times in at least N projects in the committer-only dataset.

We repeated the vocabulary selection process for the committer-only dataset. Here the common sets keep the same average percentages of frequency of tokens in projects as the full dataset common sets. For committer-only data, *CommonSet 1* is size of 115, corresponding to *M* = 30 and *N* = 30. *(CommonSet 2)* has 533 words matching the choice of *M* = 5 and *N* = 30. *CommonSet 3* consists of 1008 words, according to the choice of *M* = 30 and *N* = 10. The 3026 words in *CommonSet 4* are selected for *M* = 5 and *N* = 10. Fig 7 shows the LM performance on these word sets in the committer-only dataset. As the ratio of known tokens increases, and more infrequent words are joined, the prediction performance improves. This trend appears linear in SpeakingP and super-linear in SpokenP. This implies that infrequent tokens play a more crucial role in predicting who is addressed than predicting who is speaking.

### Project-level validation

Like other neural network models, our LMs have millions of weights and parameters. Most of them are trainable based on data input. In this section, we assess the robustness of our LMs from the perspective of project-based corpus subsets. Until now, in the models above, we disregarded project information when splitting the whole data into training and test sets, thus both sets consist of posts from different projects all mixed together. But we also wanted to know if the performance on our tasks can carry across projects (cross project prediction). To accomplish this, we ran two related experiments: status prediction within only one project, and status prediction with the test data from a project held out of the training set. The training and test size ratio is still 70 : 30.

Table 3 shows the cross project AUC for both scenarios. Limited by the project difference and the goal of comparison, we only tested on 12 projects whose @-mention post count is larger than 2, 000 in SpeakingP. We matched the training and test sizes for cross project and within project validation in SpeakingP to be the same as the corresponding set sizes in SpokenP, in order to make the results comparable.

**Table 3:** Project-level validation for both scenarios.

Data	Task	SpeakingP downsample	SpokenP
Full Data	Cross project	75.53 (5.54)	66.50 (7.71)
	Within project	69.10 (10.76)	67.43 (8.90)
	Original	84.20	81.06
Committer Data	Cross project	62.77 (5.32)	60.97 (5.76)
	Within project	61.86 (9.16)	59.49 (9.89)
	Original	75.16	75.04

The first number in the cell is the mean of AUCs for different projects, and the number in parentheses is the standard deviation of these AUCs.

Due to the smaller data sizes available, the performance drops across the board from the “original” (full data) setting. Regardless of the cross- vs. within-project distinction, we see that the AUC for SpeakingP remains higher than that of SpokenP. In general, we note that the variances in the within-project performance are greater (far greater in the case of SpeakingP than with SpokenP). Clearly, projects have varying linguistic norms; some projects may have a social norm of being consistently deferential to high-status members; others may not. Averaging data across many projects suppresses this variance. This greater within-project variance also indicates that the slightly lower within-project performance in some cases is just an artifact, unlikely to be of interest. Overall, the data suggests that there’s not a notable difference between within- and cross-project prediction performance.

### Tagee recommendation

We remind the reader that the training goal of the sDAE-like model is to predict the omitted @-mention name in the post. This naturally leads to another task: using only the text in the communication, to predict *who should be @-mentioned*. Since we can mine the entire thread, we know who responded and who didn’t; we can thus harvest abundant training data relating a post’s textual content with the respondent individuals. If such a model could be trained, it would be a useful tool to help tag responsive people, thus helping to create richer and more useful discussions. New developers, with limited social connections, might find this specially helpful.

We split developers into types according to their relationship within a certain thread: responsive and irresponsive, tagged and untagged. A developer who has been @-mentioned is tagged, otherwise untagged for this post. Our key idea about responsiveness is: developers who join in the thread after they are @-mentioned are responsive, and thus were appropriately tagged, in this discussion. If responsive, they are arguably at least trying to be helpful, and thus are perhaps a good person to call on in the future.

Since this task focuses on the recommendation quality, we chose two performance evaluation metrics. The first is the *top-K accuracy*, which is the percentage of times the target is recommended within a top-K result, defined as follows (with total possible targets *n*):

$$Top-K\;Accuracy=\frac{\sum 1(target\in \text{top}-\text{k}\;\text{prediction})}{n}$$ (1)

The second is the mean reciprocal rank (MRR). MRR is a normalized measure of the inverse ranks of the relevant observations among the queries. MRR has a range of [0, 1], where higher MRR indicates a better prediction. The definition is as follows, where *Q* is the number of queries, and *rank*_*i*_ is the rank of the first relevant query result:

$$MRR=\frac{1}{\left|Q\right|}\sum_{i=1}^{\left|Q\right|}\frac{1}{rank_{i}}$$ (2)

Table 4 shows the performance of the sDAE-like recommendation model for multiple experiments. The first is a direct @-mention prediction based on the 163, 789 posts which contain at least one @-mention. The top-1 accuracy is 18.97%, with Top-3 accuracy of 26.86%. When we randomly downsampled to 80% of the whole data from the table, the accuracies were not affected significantly. We see that the top-3 accuracies and the MRRs track closely. The top-1 accuracy almost stays the same, while the top-3 accuracy and top-5 accuracy drop by 2%.

**Table 4:** Recommendation accuracy results on the full dataset.

Test	Test size	Accuracy			MRR
		Top-1	Top-3	Top-5	
Tagged developer	32758	18.97%	26.86%	30.91%	0.2476
Tagged developer downsample	24141	17.10%	24.63%	27.13%	0.2286
Tagged & responsive	3600	25.28%	37.40%	44.64%	0.3440
	3600 (retrained)	28.30%	41.28%	48.57%	0.3786
Baseline predictor 1 (Indegree)	32758	0.586%	1.73%	2.86%	0.00
Baseline predictor 2 (Commit count)	32758	0.182%	0.541%	0.891%	0.00

For comparison purposes, we created two baseline predictors to validate our prediction accuracy. The first, denoted “Baseline predictor 1”, is based on the in-degree of a developer in the @-mention network. New developers, e.g., can see who is @-mentioned across posts, and may call highly mentioned people in the project since they have been previously asked for help. The second baseline predictor, denoted “Baseline predictor 2”, is based on the commit count of developers, which is visible to all in the project. It stands to reason that highly productive people are more likely to be mentioned because of their experience. Table 4 shows that our tool easily dominates these two baselines.

We also tested the hypothesis that tagged and responsive developers (those who respond to the thread in which they are tagged) are more likely to be the correct people to tag, *vs*. those that are not tagged or responsive; *i.e*., the individual who is observed as @-mentioned is not the correct person to call—the call should have been made to another (responsive) individual. In this test, the target is *each individual* who has posted in the thread, *after* a post mentions him/her within a thread. We sampled 3,600 posts by tagged and responsive developers (20% of the all posts by such developers) as a test set and trained on posts that are not marked both tagged and responsive. This model is then retrained on other tagged and responsive posts and tested on the same test set again (Table 4). Our results show that our modified model predicts tagged and responsive developers much better than the original: the top-1 accuracy is 25.28% and the top-3 accuracy is 37.40%, before retraining. After retraining, the result is even stronger, since similar posts are added into the training set. The top-K accuracies increase by 3–5%, and the MRR also has appreciable increase of 5%. This implies that our sDAE tool can better predict individuals who are responsive to a given issue, even if these individuals were not initially @-mentioned.

For the committer-only dataset the results are in Table 5, and the patterns are similar, except for the tagged and responsive developer prediction. For the latter, the accuracies show no difference. The retraining also does not help much. This suggests that within the committer-only dataset, our model has no preference for responsiveness, perhaps owing to the fact that committers are much more responsive than non-committers in general.

**Table 5:** Recommendation accuracy on the committer-only dataset.

Test	Test size	Accuracy			MRR
		Top-1	Top-3	Top-5	
Tagged developer	19916	16.22%	28.30%	34.84%	0.2523
Tagged developer downsample	15933	13.07%	24.91%	31.46%	0.2201
Tagged & responsive	2410	17.22%	28.09%	35.35%	0.2573
	2410 (retrained)	18.88%	30.00%	35.89%	0.2720
Baseline predictor 1 (Indegree)	19916	0.775%	2.299%	3.794%	0.00
Baseline predictor 2 (Commit count)	19916	0.592%	1.776%	2.946%	0.00

**Fig 8:**
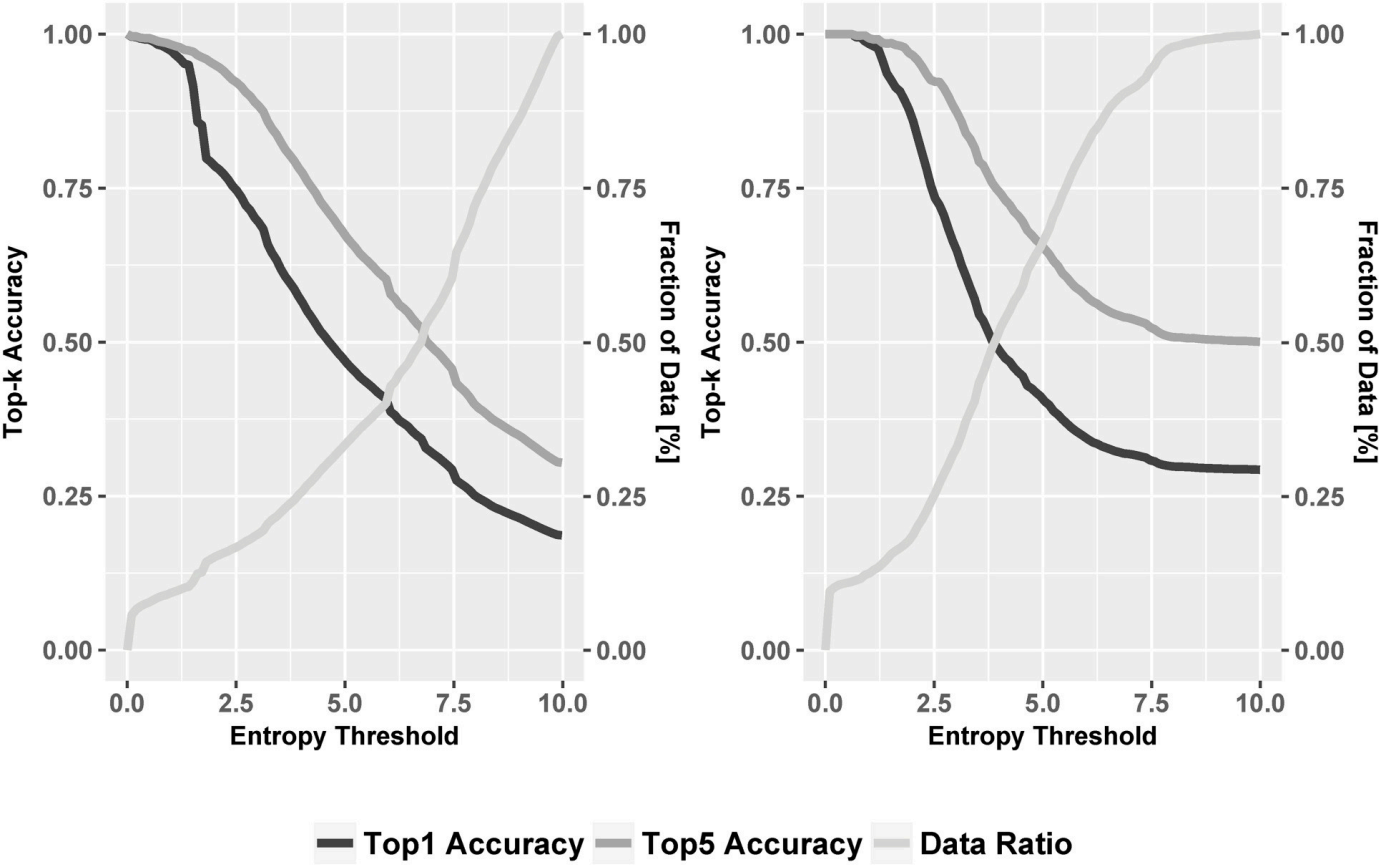
Line plot of entropy influence on accuracy (left: Row 1; right: Row 4 from Table 4).

Finally, we check if the model’s “confidence” of the predicted individual relates to accuracy. For this, we calculate the entropy of the predicted probability distribution of individuals, coming out of the predictor’s dense layer for every post. If the prediction is very skewed towards a few individuals, it will have a lower entropy than a prediction that distributes the probability mass uniformly across all individuals. So a skewed (lower-entropy) prediction could be considered more “confident”. By choosing varying entropy levels as thresholds, we selected posts whose entropy are lower than the threshold, and calculated the accuracies within this sub-dataset. Fig 8 shows the relationship between entropy of the developer score and the accuracy of the sDAE-like model, for both Row 1 (left) and Row 4 (right) from Table 4.

When the entropy increases, the model gets more uncertain about the prediction, which leads to a top-k accuracy drop. The entropies for posts are basically uniformly distributed, so the most light curve rises more or less uniformly. The maximum entropy for Row 1 (left) is higher. For the left hand size curve, Overall, the top-1 accuracy suffers sharp decrease from 90% when entropy is around 2 bits, corresponding to 12% of the data. This may imply that that fraction of the data has less ambiguous @-mention candidates that can be well predicted. For entropy larger than 2.0, both accuracy lines decline with similar slopes. On the right curve, accuracy remains high for somewhat higher thresholds, and the lowest accuracy is higher, reflecting the lower level of the entropy maximum.

Using the Row 1 configuration, it is noteworthy that high levels of accuracy can be achieved for about 12% of the cases. This means we can provide *extremely* accurate suggestions. In such cases, if no one is tagged, or if the guess from the learned model is other than the person actually tagged, a tool might offer suggestions on who else should be tagged. This could facilitate task-oriented social interactions.

## Conclusion

Modern tools such as GitHub support social coding, where developers interact via asynchronous textual media to develop software collaboratively. There is abundant textual communication data, which includes social tagging (@-mentions) where people are specifically addressed, to request their attention to technical discussions. We describe a series of experiments exploring the use of this textual data to identify the status of both speakers and those being spoken to, using model language models, and stacked de-noising to convert the text into continuous vector representations. We find good performance, and then examine closely the features of the text that might be leading to the strong performance. We experimentally discount non-content factors such as length, syntactic markers, etc, and find evidence suggesting that the semantic content is the primary factor behind our models’ good performance. In a follow-on experiment, we find that even the identity of *who should be tagged* can also be found with a reasonable level of accuracy. Finally, we note our work addresses a purely scientific question concerning the socio-linguistics of tagged exchanges in technical communities. We do acknowledge the possibility of improving on our task performance using other features, such as related source, prior social connections between tagger and (potential) tagee etc; we leave this for future work.

## Supporting information

S1 Code All the code we used for modelling and analysis is here. (ZIP)

S1 Data Word lists. Common words sets 1-4.
(RAR)

S2 Data Raw comment data in issue section. (ZIP)

S3 Data Raw commit history data. (RAR)

## References

[1] Storey MA, Ryall J, Singer J, Myers D, Cheng LT, Muller M. How software developers use tagging to support reminding and refinding. IEEE Transactions on software engineering. 2009;35(4):470–483. 10.1109/TSE.2009.15

[2] Issue with WebSocket through SSL; 2018. Available from: https://github.com/puma/puma/issues/1189.

[3] Mockus A, Fielding RT, Herbsleb JD. Two case studies of open source software development: Apache and Mozilla. ACM Transactions on Software Engineering and Methodology (TOSEM). 2002;11(3):309–346. 10.1145/567793.567795

[4] Bird C, Nagappan N, Murphy B, Gall H, Devanbu P. Don’t touch my code!: examining the effects of ownership on software quality. In: Proceedings of the 19th ACM SIGSOFT symposium and the 13th European conference on Foundations of software engineering. ACM; 2011. p. 4–14.

[5] Vincent P, Larochelle H, Lajoie I, Bengio Y, Manzagol PA. Stacked Denoising Autoencoders: Learning Useful Representations in a Deep Network with a Local Denoising Criterion. J Mach Learn Res. 2010;11:3371–3408.

[6] Dabbish L, Stuart C, Tsay J, Herbsleb J. Social coding in GitHub: transparency and collaboration in an open software repository. In: Proceedings of the ACM 2012 conference on Computer Supported Cooperative Work. ACM; 2012. p. 1277–1286.

[7] Tsay J, Dabbish L, Herbsleb J. Let’s talk about it: evaluating contributions through discussion in GitHub. In: Proceedings of the 22nd ACM SIGSOFT international symposium on foundations of software engineering. ACM; 2014. p. 144–154.

[8] McDonald N, Goggins S. Performance and participation in open source software on github. In: CHI’13 Extended Abstracts on Human Factors in Computing Systems. ACM; 2013. p. 139–144.

[9] Zhang Y, Wang H, Yin G, Wang T, Yu Y. Social media in GitHub: the role of @-mention in assisting software development. Science China Information Sciences. 2017;60(3):032102 10.1007/s11432-015-1024-6

[10] Zhang Y, Wang H, Yin G, Wang T, Yu Y. Exploring the use of @-mention to assist software development in github. In: Proceedings of the 7th Asia-Pacific Symposium on Internetware. ACM; 2015. p. 83–92.

[11] Jeong G, Kim S, Zimmermann T. Improving bug triage with bug tossing graphs. In: Proceedings of the the 7th joint meeting of the European software engineering conference and the ACM SIGSOFT symposium on The foundations of software engineering. ACM; 2009. p. 111–120.

[12] Chen L, Wang X, Liu C. An Approach to Improving Bug Assignment with Bug Tossing Graphs and Bug Similarities. JSW. 2011;6(3):421–427.

[13] Yu Y, Wang H, Yin G, Ling CX. Who should review this pull-request: Reviewer recommendation to expedite crowd collaboration. In: Software Engineering Conference (APSEC), 2014 21st Asia-Pacific. vol. 1. IEEE; 2014. p. 335–342.

[14] Yu Y, Wang H, Yin G, Wang T. Reviewer recommendation for pull-requests in GitHub: What can we learn from code review and bug assignment? Information and Software Technology. 2016;74:204–218. 10.1016/j.infsof.2016.01.004

[15] Bird C, Gourley A, Devanbu P, Swaminathan A, Hsu G. Open borders? immigration in open source projects. In: Proceedings of the Fourth International Workshop on Mining Software Repositories. IEEE Computer Society; 2007. p. 6.

[16] Kavaler D, Sirovica S, Hellendoorn V, Aranovich R, Filkov V. Perceived language complexity in GitHub issue discussions and their effect on issue resolution. In: Proceedings of the 32nd IEEE/ACM International Conference on Automated Software Engineering. IEEE Press; 2017. p. 72–83.

[17] Adam H, Galinsky AD. Enclothed cognition. Journal of Experimental Social Psychology. 2012;48(4):918–925. 10.1016/j.jesp.2012.02.008

[18] Slepian ML, Ferber SN, Gold JM, Rutchick AM. The cognitive consequences of formal clothing. Social Psychological and Personality Science. 2015;6(6):661–668. 10.1177/1948550615579462

[19] Labov W. The social stratification of English in New York city. Cambridge University Press; 2006.

[20] Deckert SK, Vickers CH. An introduction to sociolinguistics: Society and identity. A&C Black; 2011.

[21] Kacewicz E, Pennebaker JW, Davis M, Jeon M, Graesser AC. Pronoun use reflects standings in social hierarchies. Journal of Language and Social Psychology. 2014;33(2):125–143. 10.1177/0261927X13502654

[22] Dino A, Reysen S, Branscombe NR. Online interactions between group members who differ in status. Journal of Language and Social Psychology. 2009;28(1):85–93. 10.1177/0261927X08325916

[23] PyGitHub; 2018. Available from: https://github.com/PyGithub/PyGithub.

[24] GitHub. Mastering Issues(GitHub Guides); 2018. Available from: https://guides.github.com/features/issues/.

[25] Zero-inflated model; 2019. Available from: https://en.wikipedia.org/wiki/Zero-inflated_model.

[26] Project N. NLTK tokenize package; 2017. Available from: http://www.nltk.org/api/nltk.tokenize.html.

[27] Shen Y, Rong W, Jiang N, Peng B, Tang J, Xiong Z. Word Embedding based Correlation Model for Question/Answer Matching. CoRR. 2015;abs/1511.04646.

[28] Hu B, Lu Z, Li H, Chen Q. Convolutional Neural Network Architectures for Matching Natural Language Sentences. In: Ghahramani Z, Welling M, Cortes C, Lawrence ND, Weinberger KQ, editors. Advances in Neural Information Processing Systems 27. Curran Associates, Inc.; 2014. p. 2042–2050. Available from: http://papers.nips.cc/paper/5550-convolutional-neural-network-architectures-for-matching-natural-language-sentences.pdf.

[29] Pennington J, Socher R, Manning CD. GloVe: Global Vectors for Word Representation. In: Empirical Methods in Natural Language Processing (EMNLP); 2014 p. 1532–1543. Available from: http://www.aclweb.org/anthology/D14-1162.

[30] Ghosh S, Chollet M, Laksana E, Morency LP, Scherer S. Affect-lm: A neural language model for customizable affective text generation. arXiv preprint arXiv:170406851. 2017;.

[31] Markel M. Technical Communication. Bedford/St. Martin’s; 2014 Available from: https://books.google.com/books?id=MTUSBgAAQBAJ.

[32] ThoughtCo. Closed Class (Words); 2018. Available from: https://www.thoughtco.com/what-is-closed-class-words-1689856.

[33] Zeman D. Closed Class list; 2011. Available from: https://mailman.uib.no/public/corpora/2011-November/014318.html.

[34] Montemurro MA, Zanette DH. Universal Entropy of Word Ordering Across Linguistic Families. PLOS ONE. 2011;6(5):1–9. 10.1371/journal.pone.0019875 PMC3094390 21603637

[35] Most common words in English; 2018. Available from: https://en.wikipedia.org/wiki/Most_common_words_in_English.

[36] Perc M. Evolution of the most common English words and phrases over the centuries. Journal of The Royal Society Interface. 2012 10.1098/rsif.2012.0491 PMC3481586 22832364

